# Novel Pathways of Ponatinib Disposition Catalyzed By CYP1A1 Involving Generation of Potentially Toxic Metabolites[Fn FN3]

**DOI:** 10.1124/jpet.117.243246

**Published:** 2017-10

**Authors:** De Lin, Rumen Kostov, Jeffrey T.-J. Huang, Colin J. Henderson, C. Roland Wolf

**Affiliations:** Division of Cancer Research, Jacqui Wood Cancer Centre (D.L., C.J.H., C.R.W.), Molecular & Cellular Medicine (R.K.), and Biomarker & Drug Analysis Core (J.T.-J.H.), School of Medicine, Ninewells Hospital, University of Dundee, Dundee, United Kingdom

## Abstract

Ponatinib, a pan-BCR-ABL tyrosine kinase inhibitor for the treatment of chronic myeloid leukemia (CML), causes severe side effects including vascular occlusions, pancreatitis, and liver toxicity, although the underlying mechanisms remain unclear. Modifications of critical proteins through reactive metabolites are thought to be responsible for a number of adverse drug reactions. In vitro metabolite screening of ponatinib with human liver microsomes and glutathione revealed unambiguous signals of ponatinib-glutathione (P-GSH) adducts. Further profiling of human cytochrome P450 (P450) indicated that CYP1A1 was the predominant P450 enzyme driving this reaction. P-GSH conjugate formation paralleled the disappearance of hydroxylated ponatinib metabolites, suggesting the initial reaction was epoxide generation. Mouse glutathione *S*-transferase p1 (mGstp1) further enhanced P-GSH adduct formation in vitro. Ponatinib pharmacokinetics were determined in vivo in wild-type (WT) mice and mice humanized for CYP1A1/2 and treated with the CYP1A1 inducers 2,3,7,8-tetrachlorodibenzodioxin or 3-methylcholanthrene. Ponatinib exposure was significantly decreased in treated mice compared with controls (7.7- and 2.2-fold for WT and humanized CYP1A1/2, respectively). Interestingly, the P-GSH conjugate was only found in the feces of CYP1A1-induced mice, but not in control animals. Protein adducts were also identified by liquid chromatography–tandem mass spectrometry analysis of mGstp1 tryptic digests. These results indicate that not only could CYP1A1 be involved in ponatinib disposition, which has not been previously reported, but also that electrophilic intermediates resulting from CYP1A1 metabolism in normal tissues may contribute to ponatinib toxicity. These data are consistent with a recent report that CML patients who smoke are at greater risk of disease progression and premature death.

## Introduction

Ponatinib (Iclusig) is an orally available pan-BCR-ABL tyrosine kinase inhibitor that has been approved for treatment of resistant chronic myeloid leukemia (CML) and Philadelphia chromosome-positive acute lymphoblastic leukemia (Zhou et al., 2011; Cortes et al., 2012; Razzak, 2013; Miller et al., 2014). Ponatinib was structurally designed with a carbon-carbon triple-bond linkage to target the T315I mutation (BCR-ABL^T315I^), which confers resistance to other approved tyrosine kinase inhibitors (O’Hare et al., 2009; Huang et al., 2010). Ponatinib has high efficacy against multiple resistant forms, including T315I mutation in a phase I clinical trial of ponatinib in refractory Philadelphia chromosome-positive acute lymphoblastic leukemia (Cortes et al., 2012) and T315I tyrosine kinase inhibitor–resistant CML cells (O’Hare et al., 2009). However, serious side effects have been reported; thrombocytopenia (in 37% of patients) and neutropenia (19%) are the most common hematologic events of ponatinib treatment (Cortes et al., 2012). Hepatotoxicity has also been reported, including alanine aminotransferase and aspartate aminotransferase elevation in patients in clinical trials (10.5% and 8.2%, respectively). Cardiovascular toxicity, including both arterial and venous thromboembolism and severe systemic hypertension, and dose-limiting toxic effects limit its broad application in clinic (Frankfurt and Licht, 2013; ARIAD Pharmaceuticals, Cambridge, MA, 2014 Iclusig package insert). However, the mechanism of ponatinib toxicity remains unclear.

The metabolic profile of ponatinib has been evaluated in preclinical studies. The disposition of ponatinib is a result of both esterase/amidase activity to an inactive carboxylic acid (AP24600) and metabolism by the cytochrome P450 (P450)–dependent monooxygenase system [ARIAD Pharmaceuticals, 2013 Iclusig (ponatinib) tablets for oral use: prescribing information (http://wwwaccessdatafdagov/drugsatfda_docs/label/2013/203469s007s008lblpdf); Narasimhan et al., 2013]. Ponatinib is metabolized by P450 CYP3A4 (and to a lesser extent by CYP3A5, CYP2C8, and CYP2D6) to *N*-oxide and *N*-desmethyl metabolites, the latter being 4-fold less potent than ponatinib. Although the hydrolysis of ponatinib has been reported to be the major pathway of disposition, a recent study showed that concurrent therapy with ponatinib and ketoconazole (a CYP3A4 inhibitor) significantly increased the ponatinib area under the curve (AUC) AUC_0–∞_ and AUC_0–_*_t_* values and the *C*_max_ values (Narasimhan et al., 2013).

Levels of drug exposure, as well as the generation of chemically reactive metabolites, can play a major role in drug side effects (Baillie, 2006; Kalgutkar and Dalvie, 2015). To investigate this possibility, we carried out a detailed analysis of ponatinib metabolism in a panel of purified recombinant human P450 enzymes. In addition to identifying CYP3A4 as a major pathway of ponatinib disposition (Narasimhan et al., 2013), we have also found that CYP1A1, an inducible enzyme in tissues such as lung and lung tumors, is highly active toward this compound. Intriguingly, metabolism by CYP1A1 resulted in the formation of an electrophilic metabolite, which formed a glutathione (GSH) conjugate in a reaction catalyzed by the glutathione *S*-transferases (GSTs). The significance of these data in relation to the efficacy and side effects of ponatinib is discussed.

## Materials and Methods

### 

#### Chemicals and Reagents.

All reagents, unless otherwise stated, were purchased from Sigma-Aldrich (Poole, United Kingdom). NADPH was obtained from Melford Laboratories (Ipswich, United Kingdom), ponatinib was purchased from LC Laboratories (Woburn, MA), and 2,3,7,8-tetrachlorodibenzodioxin (TCDD) was purchased from Toronto Research Chemicals (Toronto, Canada). The Nanosep centrifugal device with Omega ultrafiltration membrane molecular weight cutoff of 10 kDa was obtained from Pall Corporation (East Hills, NY).

#### Animal Lines and Husbandry.

The generation and characterization of hCYP1A1/1A2 mice will be described elsewhere (manuscript in preparation). All animals were maintained under standard animal house conditions, with free access to food (RM1 diet; Special Diet Services, Essex, United Kingdom) and water, and a 12-hour light/12-hour dark cycle. All animal work was carried in accordance with the Animal Scientific Procedures Act (1986), as amended by European Union Directive 2010/63/EU, and after local ethical review.

#### Subcellular Fractionation.

Livers were harvested, excised, and snap frozen in liquid nitrogen for storage at −80°C until processing. Briefly, a small piece (∼200 *µ*g) in three volumes of KCl-phosphate buffer (10 mM KHPO_4_, 20 mM EDTA, and 150 mM KCl, pH 7.4) was homogenized (POLYTRON PT 2100; Kinematica, Lucerne, Switzerland). The homogenate was centrifuged for 80 minutes at 100,000*g* (30,000 rpm in an F50L rotor) at 4°C. After removal of the fatty layer, the resultant supernatant was centrifuged for 1 hour at 100,000*g* (30,000 rpm in an F50L rotor; Thermo Scientific, Perth, UK). After ultracentrifugation, the supernatant (cytosolic fraction) was retained and the pellet (microsomal fraction) was resuspended in KCl buffer containing 0.25 M sucrose. The protein content of the microsomal and cytosolic fractions was quantified by bicinchoninic acid assay (Thermo Scientific).

#### Glutathione-Affinity Chromatography.

The recombinant mouse glutathione *S*-transferase p1 (mGstp1) and GST mixture from liver cytosol (mouse and human) were purified using a GSTrap FF affinity column (1 ml; Amersham Biosciences AB, Uppsala, Sweden) according to the manufacturer’s protocol. Briefly, the column was equilibrated with five column volumes of binding buffer (phosphate-buffered saline, pH 7.3). The sample was filtered through a 0.45 *µ*m filter and loaded onto the column at a flow rate of 1 ml/min, followed by washing with five columns of binding buffer, and then the bound protein was eluted by adding five column volumes of elution buffer (50 mM Tris-HCl, 10 mM reduced GSH, pH 8.0, at a flow rate of 1 ml/min). The eluted protein was collected and analyzed by SDS-PAGE. The proteins were concentrated and buffer exchanges were performed using a 10 kDa Nanosep centrifugal device (previously conditioned by wetting with 0.5 ml of distilled water and followed by drying by centrifugation at 14,000*g* for 10 minutes at 4°C) to 50 mM Tris-HCl, pH 7.4. The retained protein residues were quantitatively dislodged from the membranes by using 100 *µ*l aliquots of 50 mM Tris-HCl, pH 7.4. Protein concentration was then quantified by a bicinchoninic acid assay using the Pierce BCA Protein Assay Kit (Thermo Fisher Scientific, Somerset, NJ) according to the manufacturer’s instructions.

#### In Vitro Metabolism.

All incubations were performed at 37°C and 300 rpm in a thermomixer. Human cDNA-expressed P450 isoenzymes coexpressed with human NADPH P450 oxidoreductase (see Supplemental Fig. 1 for CYP:oxidoreductase ratio data) were carefully thawed on ice prior to the experiment. Ponatinib (50 *µ*M with 0.25% dimethylsulfoxide) was mixed with human P450 isoenzymes (100 nM) in 100 mM potassium phosphate buffer (pH 7.4) containing 3.3 mM MgCl_2_ supplemented with or without GSH and a GST mixture from mouse or human liver cytosol. After 5-minute preincubation at 37°C, the incubation reactions were initiated by addition of a NADPH regenerating system (final concentration: 1.3 mM NADPH, 4 mM glucose-6-phosphate, and 2 unit/ml glucose-6-phosphate dehydrogenase). After 5–120 minutes, an aliquot of 100 *µ*l of reaction mixture was quenched with same volume of cold acetonitrile containing 400 ng/ml of triazolam as the internal standard. The samples were then centrifuged at 3000*g* for 55 minutes at 4°C. An aliquot of the supernatant fraction was analyzed by liquid chromatography (LC)–tandem mass spectrometry (MS), i.e., LC-MS/MS.

#### In Vivo Studies.

All animal work was carried out on 8- to 12-week-old male mice. 3-Methylcholanthrene (3-MC) was suspended in corn oil at 4 mg/ml for administration by intraperitoneal injection at 40 mg/kg (10 *μ*l/g body weight) daily for 3 days for hCYP1A1/2 mice. TCDD was suspended in corn oil at 1 *µ*g/ml for administration by intraperitoneal injection at 10 *µ*g/kg (10 *μ*l/g body weight) daily for 3 days for wild-type (WT) mice. For pharmacokinetic (PK) analyses, ponatinib was first dissolved in 25 mM citrate buffer (pH 2.5) at a concentration of 4 mg/ml for immediate administration by oral gavage at a final dose of 40 mg/kg. For blood sample collection, 10 *μ*l of whole blood was withdrawn from the tail vein at the indicated time points. Samples were immediately added to a tube containing heparin solution (10 *μ*l, 15 IU/ml), put into liquid nitrogen, and then stored at −80°C until processing. On the day of analysis, acetonitrile (80 *µ*l containing 200 ng/ml triazolam) was added to thawed samples, which were shaken for 15 minutes, centrifuged for 10 minutes at 16,000*g*, and analyzed. Plasma ponatinib concentrations were determined by an internal standard LC-multiple reactions monitoring (MRM) method using calibration standards prepared in blank mouse plasma. Feces were collected in Tecniplast mouse metabolic cages (Tecniplast, Leicester, UK); mice were housed singly with free access to food and water for up to 24 hours after administration of ponatinib dose. To measure fecal GSH ponatinib metabolites each sample was diluted by water (1:3 v/v) and homogenized with a mortar and pestle. An aliquot of 0.25 g was extracted with 1 ml of 80% methanol for 30 minutes on a vortex evaporator (Labconco, Kansas City, MO). After centrifugation (30 minutes at 4000*g*), the solvent of a 800 *μ*l aliquot of the supernatant was removed by speed vacuum, followed by reconstitution in 500 *μ*l methanol and water (50/50, v/v), and analyzed by LC-MRM.

#### Western Immunoblotting Analyses.

Liver microsomal samples were adjusted to 1 mg/ml in LDS Lithium Dodecyl Sulfate sample buffer (Life Technologies) and subjected to SDS-PAGE and immunoblotting as previously described (MacLeod et al., 2015). Primary antibodies were used as follows: anti-CYP1A1 (1:1000; AB1258; Millipore, Watford, United Kingdom) and Cyp1a (1:1000) (Forrester et al., 1992). Anti-GRP78 (1:1000; ab21685; Abcam (Cambridge, UK)) was used as the loading control. Immunoreactive proteins were detected using polyclonal goat anti-rabbit or anti-mouse horseradish peroxidase immunoglobulins as secondary antibodies (Dako, Ely, United Kingdom) and were visualized using Immobilon chemiluminescent substrate (Millipore) and a Fujifilm LAS-3000 mini-imaging system (Fujifilm UK Ltd., Bedford, United Kingdom).

####  LC-MS-MRM Analysis.

Analysis of in vitro incubation and in vivo blood PK samples was carried out by ultra-performance LC-MS/MS using the Waters Acquity UPLC (Micromass, Manchester, United Kingdom) and Micromass Quattro Premier Mass Spectrometer with electrospray ionization. The chromatography was performed using a C18 column (Kinetex 1.7 *µ* 100 A; 50 × 2.1 mm; Phenomenex, Macclesfield, United Kingdom) at a temperature of 45°C with mobile phases of 0.1% formic acid (A) and acetonitrile and 0.1% formic acid (B). A gradient at a flow rate of 0.5 ml/min was run over 3 minutes as follows: 0–0.5 minutes, 95% A; 0.5–0.75 minutes, 95%–67.5% A; 0.75–1.50 minutes, 67.5% A, 1.50–2.00 minutes, 67.5%–50% A; 2.00–2.50 minutes, 50%–5% A; and then returning to the initial conditions for a final 0.5 minutes. The cone voltage and collision energy were optimized for each substrate and MRM data were acquired (Supplemental Table 1).

#### liquid chromatography eletcrospray ionization/multi-stage mass spectrometry (LC-MS-MS^n^) Analysis.

The LC-MS/MS system consisted of a Waters Alliance 2690 high-performance LC system (Waters, Milford, MA) and a LTQ-Orbitrap XL mass spectrometer (Thermo Fisher Scientific) with an electrospray ionization interface. The chromatography was performed on a XB-18 column (Kinetex 50 × 2.1 mm, 2.6 *µ*m particle size; Phenomenex) at a temperature of 40°C. Analytes were eluted from the column with 0.1% formic acid in water (A) and 0.1% formic acid in acetonitrile (B). A gradient at a flow rate of 0.4 ml/min was run over 18 minutes as follows: 0–0.5 minutes, 95%–80% A; 0.5–10 minutes, 80%–5% A; 10–12.5 minutes, 5% A; 12.5–14 minutes, 5%–95% A; and 14–18 minutes, 95% A. The mass spectrometer was tuned to optimal conditions for ponatinib and was operated in a data-independent acquisition mode, which consisted of two scan events: a survey full scan (150–600 m/z) at a resolution of 30 K Orbitrap and multi-stage mass spectrometry (MS^n^) scan at an ion trap. The product ions were generated by collision-induced dissociation mode.

#### Bioactivation of Ponatinib by Human Recombinant CPY1A1 in the Presence of mGstp1.

To generate protein adducts of oxidative ponatinib metabolites with mGstp1, incubations were conducted with a reaction mixture of human recombinant CYP1A1 (100 nmol) in *Escherichia coli* membrane, 10 *µ*g/ml mGstp1, and 50 *µ*M ponatinib (0.5 *µ*l 20 mM in acetonitrile) as substrate, in a final volume of 200 *µ*l 100 mM potassium phosphate buffer (pH 7.4). The incubation was started by the addition of a NADPH-regenerating system (final concentration: 1.3 mM NADPH, 4 mM glucose-6-phosophate, 2 U/ml glucose-6-phosphate dehydrogenase, and 3.3 mM potassium phosphate) and allowed to proceed at 37°C for 80 minutes. The reaction was terminated by cooling on ice, and the *E. coli* membrane containing human recombinant CYP1A1 was removed by ultracentrifugation at 41,000*g* at 4°C for 80 minutes. The supernatant was further filtered through a Pall Nanosep 10 K OMEGA (PN OD010C34) filter to remove unbound ponatinib and its metabolites. The solvent was exchanged to 50 mM ammonium bicarbonate and further concentrated to 100 *µ*l.

Aliquots of the solution (10 *µ*l) were mixed with 10 *μ*l of 4× NuPAGE lithium dodecyl sulfate loading buffer (Invitrogen, Carsbad, CA) by incubation at 95°C for 5 minutes and then subjected to SDS-PAGE on a NuPAGE Novex 10% Bis-Tris mini gel (Invitrogen NP0341BOX). After electrophoresis at a constant 180 V for 30 minutes, gels were stained with Coomassie Blue. Bands (20–25 kDa) containing mGstp1 were cut from the gel, and after removing the Coomassie Blue from the gel by adding 100 mM ammonium bicarbonate/acetonitrile (1:1, v/v) and subsequently dehydrating with 100% acetonitrile, the solvent was removed from the gel pieces under vacuum. The residue was resuspended in 0.01 *μ*g/*μ*l MS grade trypsin (Promega, Madison, WI) in 25 mM ammonium bicarbonate and incubated at 37°C overnight. Peptides were extracted by adding buffer [1:2 (v/v) 5% formic acid/acetonitrile] and evaporated by speed vacuum (Shevchenko et al., 2006). Samples were redissolved for LC-MS/MS analysis in 20 *μ*l of 5% acetonitrile containing 0.1% trifluoroacetic acid.

#### Data Analysis.

In the present study, the absolute concentrations of the metabolites in the incubation mixture could not be determined because of the lack of authentic standards. Therefore, the relative activity of the P450s was presented by comparing the concentration of the metabolite in a sample with the concentration of the same metabolite in the sample that had the highest response under the same experimental condition (Yu et al., 2010). The relative activity of the P450s toward metabolite formation could be calculated according to the ratio of the metabolite concentration to that in the sample with highest mass response. The PK parameters of in vivo data were calculated with a simple noncompartmental model using the WinNonLin software, version 4.1 (Pharsight (Certara, Cambridge, UK)) and are shown with the S.D. values. The *P* values were calculated using an unpaired *t* test; *P < 0.05, **P < 0.01, ***P < 0.001.

## Results

### 

#### Ponatinib Biotransformation and Formation of a GSH Adduct by Human Recombinant P450 Enzymes.

**Fig. 1.**
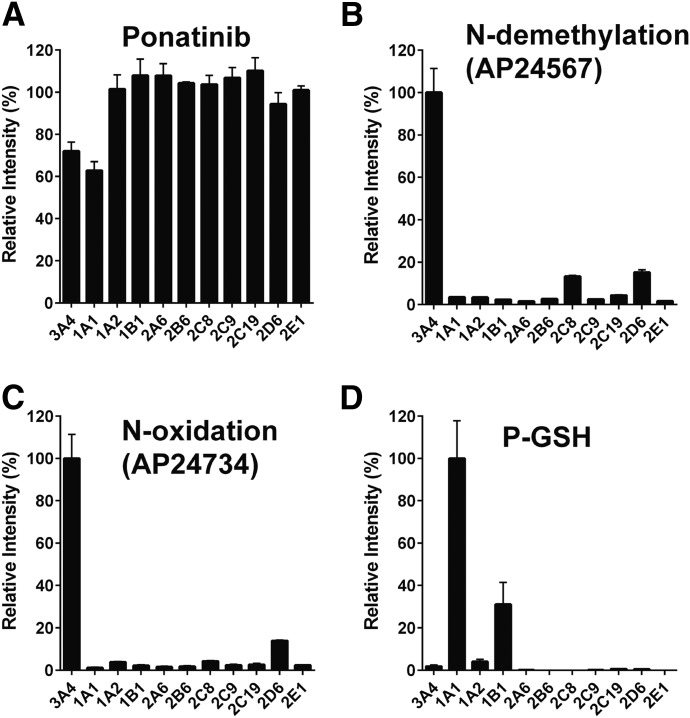
Ponatinib metabolites and GSH adduct mediated by recombinant human P450 isoforms. Recombinant human P450 isoforms were incubated with ponatinib, and parent drug, metabolites, and conjugate determined as described in *Materials and Methods*. (A) Disappearance of ponatinib. (B) Formation of *N*-demethylation (AP24567). (C) Formation of *N*-oxidation (AP24734). (D) Formation of GSH adduct (P-GSH).

**Fig. 2.**
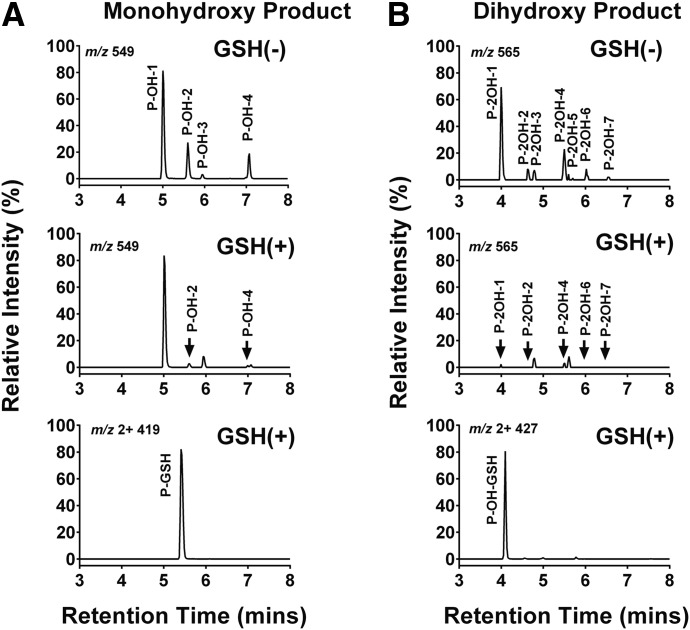
Reversed-phase LC selected ion chromatograms of ponatinib oxidation and GSH adduct mediated by CYP1A1. Ponatinib (50 *µ*M) was incubated with CYP1A1 in the presence of an NADPH regeneration system as described in *Materials and Methods*. (A) Formation of P-OH (*m/z* 549.22) (top) in the absence of GSH and mGstp1, and (middle) in the presence of 5 mM GSH and 10 *µ*g/ml mGstp1; (bottom) formation of P-GSH (*m/z*, 2+, 419.65) in the presence of 5 mM GSH and 10 *µ*g/ml mGstp1. (B) Formation of P-2OH (*m/z* 565.22) (top) in the absence of GSH and mGstp1 and (middle) in the presence of 5 mM GSH and 10 *µ*g/ml mGstp1; (bottom) Formation of P-OH-GSH (*m/z*, 2+, 427.65) in the presence of 5 mM GSH and 10 *µ*g/ml mGstp1.

The contribution of individual P450 enzymes to ponatinib biotransformation was evaluated using a panel of 11 human recombinant P450 enzymes (CYP1A1, CYP1A2, CYP1B1, CYP2A6, CYP2B6, CYPC8, CYP2C9, CYP2C19, CYP2D6, CYP3A4, and CYP2E1). Ponatinib was principally metabolized via CYP3A4 and CYP1A1, as measured by the disappearance of the parent drug (Fig. 1A). CYP3A4 had the highest activity in the formation of both the *N*-desmethyl (AP24567) and *N*-oxide (AP24734) metabolites (Fig. 1, B and C), with lesser contributions from CYP2C8 and CYP2D6. It should be noted that in the case of CYP2C8 and CYP2D6, the P450/P450 oxidoreductase ratio was considerably lower than for CYP3A4. In the event that P450 oxidoreductase is rate limiting in these samples, the actual rates of metabolism could be higher. In the case of CYP1A1, metabolism of ponatinib resulted in four different monohydroxylated products (P-OH, *m/z* 549) with retention times of 5.00 minutes (P-OH-1), 5.60 minutes (P-OH-2), 5.94 minutes (P-OH-3), and 7.07 minutes (P-OH-4), as well as a number of dihydroxylated products (see Fig. 2 and Supplemental Material for a more detailed description of the metabolites formed). The major metabolites were 2- and 4-hydroxyponatinib. These metabolites were also produced, to a lesser degree, by CYP1A2 and CYP1B1. The formation of hydroxylated metabolites could be a consequence of epoxidation reactions, a characteristic activity of CYP1A1. For this reason, we also carried out incubations in the presence of GSH. Indeed, this resulted in the formation of a ponatinib-GSH (P-GSH) conjugate. The P-OH-2 and P-OH-4 metabolites were not detected in incubations containing GSH, whereas P-OH-1 and P-OH-3 were unchanged (Fig. 2A, middle), suggesting that epoxidation had taken place at the site of these hydroxylation reactions. In addition, the formation of the dihydroxylation products was significantly reduced when GSH was added to the incubation (Fig. 2B, middle). Generation of P-GSH was not detected with any other P450 isoforms.

The MS spectrum of P-GSH revealed a molecular ion [M+2H]^2+^ of *m/z* 419.7, suggesting a GSH adduct with the addition of the sulfhydryl nucleophile to ponatinib (Supplemental Fig. 2A). Fragmentation of P-GSH molecular ions resulted in neutral loss of 75 and 129, corresponding to elimination of the glycine and pyroglutamate of GSH, respectively (Supplemental Fig. 2B), but yielded no information on the specific site of ponatinib modification. MS analysis of the fragments containing four monohydroxylated products clearly indicated that CYP1A1-mediated oxidation had occurred on the partial structure of ring A, linker B, or ring C, and that GSH conjugation had occurred on ring C (Supplemental Figs. 3–5).

#### Glutathione *S*-Transferases Catalyze the Formation of P-GSH Conjugates.

**Fig. 3.**
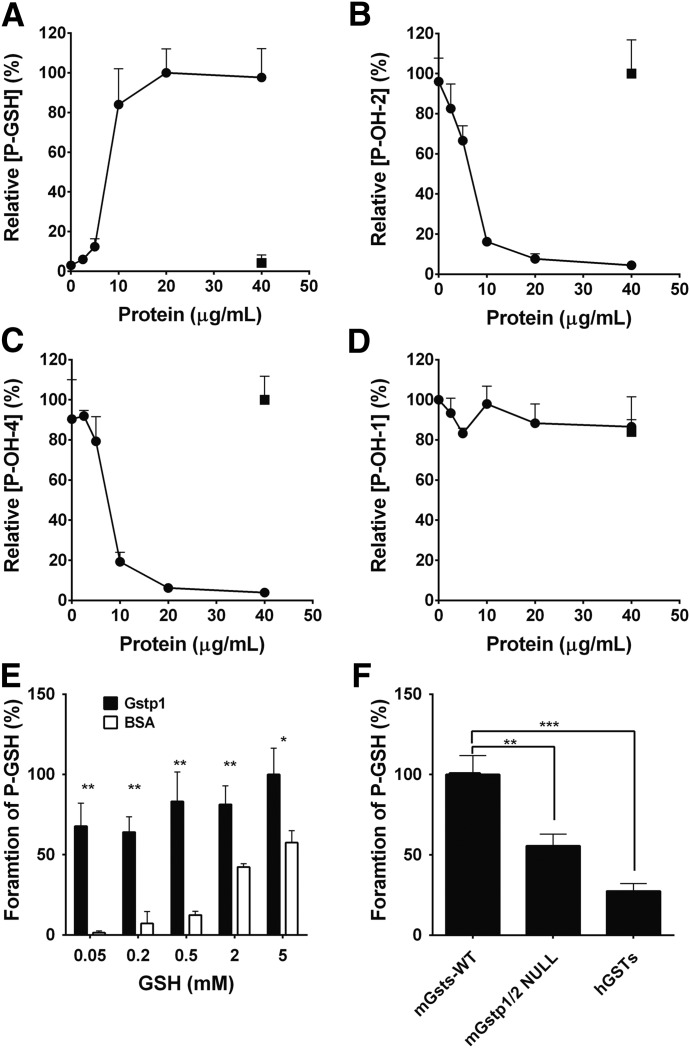
Effect of GST and GSH concentrations on the formation of monooxidation product and P-GSH. Ponatinib was incubated with CYP1A1 as described in *Materials and Methods*. (A) Formation of P-GSH, (B) formation of P-OH-2, (C) formation of P-OH-4, and (D) formation P-OH-1 in the presence of GSH and recombinant mGstp1 (black circles) or bovine serum albumin (BSA) (white square). (E) Formation of P-GSH in the presence of GSH and recombinant mGstp1 (black bar) or BSA (white bar). (F) Formation of P-GSH in the presence of GSH and GST fractions from WT, Gstp1/2 NULL mice, or human liver cytosol.

The action of electrophiles with GSH is catalyzed by GST (Hayes et al., 2005; Tew and Townsend, 2012). Therefore, we investigated this possibility through the addition of recombinant GST or GST mixtures to the incubation system. The formation of P-OH-2 and P-OH-4 was linear for at least 20 minutes and with differing concentrations of recombinant CYP1A1 protein up to 10 nM at 3 *µ*M ponatinib. Incubations were therefore routinely carried out for 20 minutes with 10 nM recombinant CYP1A1 protein. The formation of the P-GSH conjugate was significantly increased by the addition of recombinant mGstp1 protein up to 20 *µ*g/ml (Fig. 3A). The fact that this reaction was saturated at 20 *µ*g/ml suggests that the rate of hydroxylation was rate limiting in this experiment. Consistent with the data shown in Fig. 2, the formation of P-OH-2 and P-OH-4 was inversely related to the formation of the P-GSH conjugate (Fig. 3, B and C), whereas the formation of P-OH-1 was not affected by the addition of mGstp1 (Fig. 3D).

The dependence of GST-mediated P-GSH conjugate on GSH concentration is shown in Fig. 3E. At a GSH concentration of 5 mM, the rate of the mGstp1-catalyzed P-GSH conjugate formation was approximately double the rate of the nonenzymic reaction. However, at a GSH concentration of 0.05 mM the rate of the enzyme-catalyzed conjugate was increased more than 40-fold compared with the nonenzymic control (68% versus 1.4%, respectively). These differences were also reflected in an inverse manner by the formation of P-OH-2 and P-OH-4 (data not shown). Therefore, the effect of mGstp1 on the rate of P-GSH-conjugated formation is greatest at the lowest GSH concentration, consistent with a previous report that the covalent binding rate of an acetaminophen-GSH conjugate is greatest at low GSH concentrations (Rollins and Buckpitt, 1979).

To further investigate the role of GSTs in the formation of the P-GSH conjugate, Gst/GST mixtures were purified from Gstp1^WT^ and Gstp1/2^NULL^ mice and human liver through a GSTrap FF affinity column and incubated with ponatinib. Compared with the activity of the Gst mixture from mouse Gstp1^WT^ liver cytosol (100%), the formation of P-GSH was significantly reduced in both Gstp1/2^NULL^ (56%) mice indicating that, in agreement with the data obtained with the recombinant enzyme, Gstp is responsible for ∼60% of the murine hepatic conjugating activity. The activity of human liver GSTs was also lower (28%), which could be ascribed to the fact that human hepatocytes from healthy individuals do not express glutathione *S*-transferase P (GSTP) (Fig. 3F).

#### Induction of CYP1A1 Markedly Alters Ponatinib Exposure and Induces P-GSH Conjugate Excretion In Vivo.

**Fig. 4.**
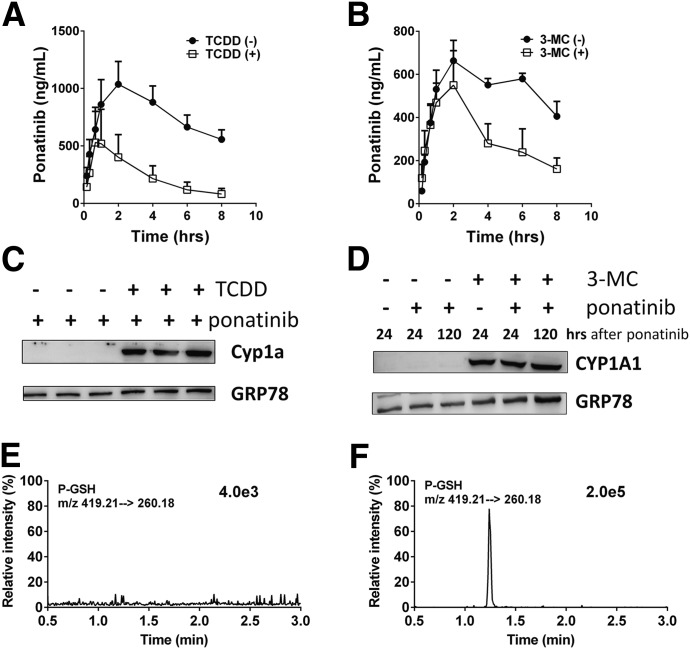
Activation of the aryl hydrocarbon receptor alters ponatinib pharmacokinetics and formation of GSH adduct in vivo. (A) PK profiles of ponatinib in WT mice treated with vehicle (circles; *n* = 3) or TCDD (squares; *n* = 3). (B) PK profiles of ponatinib in huCYP1A1/2 mice treated with vehicle (circles; *n* = 3) and 3-MC (squares; *n* = 3). (C) Western blots of hepatic microsomal fractions from WT mice dosed with either vehicle or TCDD. (D) Western blots of hepatic microsomal fractions from huCYP1A1/2 mice dosed with either vehicle or 3-MC. (E) LC-MRM chromatogram of P-GSH in feces (up to 24 hours) from ponatinib-treated humanized CYP1A1/2 mice. (F) LC-MRM chromatogram of P-GSH in feces (up to 24 hours) from ponatinib-treated humanized CYP1A1/2 mice pretreated with 3-MC. Apparent PK parameters are detailed in Table 1.

To study the contribution of CYP1A1 to the metabolism and disposition of ponatinib in vivo, WT, and humanized hCYP1A1/1A2 mice were administered the aryl hydrocarbon receptor activators TCDD or 3-MC for 3 days prior to ponatinib administration. In WT mice treated with TCDD an 87% reduction in the AUC_0–8 hours_ value and a significant decrease (62%) in the ponatinib half-life were observed (Fig. 4A; Table 1). In hCYP1A1/1A2 mice treated with 3-MC, significant reductions in both the AUC_0–8 hours_ (54%) and half-life (67%) values for ponatinib were also observed (Fig. 4B; Table 1). In addition, significant amounts of the P-GSH conjugate were detected in fecal samples from 3-MC-treated hCYP1A1/1A2 mice (Fig. 4F). This metabolite was not detectable in control (vehicle-treated) animals treated with ponatinib (Fig. 4E). The absolute level of the P-GSH conjugate formed could not be determined because relevant standards were not available.

**TABLE 1:** Pharmacokinetic parameters of ponatinib in WT mice and hCYP1A1/2 pre-treated with TCDD or 3-MC The PK parameters were calculated as outlined in *Materials and Methods*. The *P* values were calculated using an unpaired *t* test: *P ≤ 0.05; **P ≤ 0.01; ***P ≤ 0.001.

Genotype	Pretreatment	*t*_1/2_	*C*_max_	AUC_0–8 hours_	AUC_0–∞_
		*hour*	*µg/ml*	hour*µg/ml	hour*µg/ml
WT	Vehicle	6.2 ± 0.2	1,212 ± 222	7,011 ± 1,490	12,620 ± 2,230
WT	TCDD	2.4 ± 0.8**	253 ± 42**	908 ± 59**	1,044 ± 175**
hCYP1A1/1A2	Vehicle	13.9 ± 1.7	705 ± 96	4,170 ± 500	9,740 ± 2,100
hCYP1A1/1A2	3-MC	4.6 ± 0.8**	620 ± 245	1,920 ± 520*	3,650 ± 1,890*

#### Ponatinib Forms an Adduct with mGstp1 in the Presence of CYP1A1.

It has often been observed that the conjugation of electrophiles by Gstp1 also results in covalent modification of Gstp1 itself. To investigate this possibility, tryptic peptides obtained following incubations of ponatinib with CYP1A1 and Gstp1 were analyzed and two different adducts of Gstp1 were identified (Supplemental Table 2). Further MS analysis demonstrated that ponatinib-Gstp adducts had been formed on Cys-14 and Cys-47, but not on Cys-167.

## Discussion

**Fig. 5.**
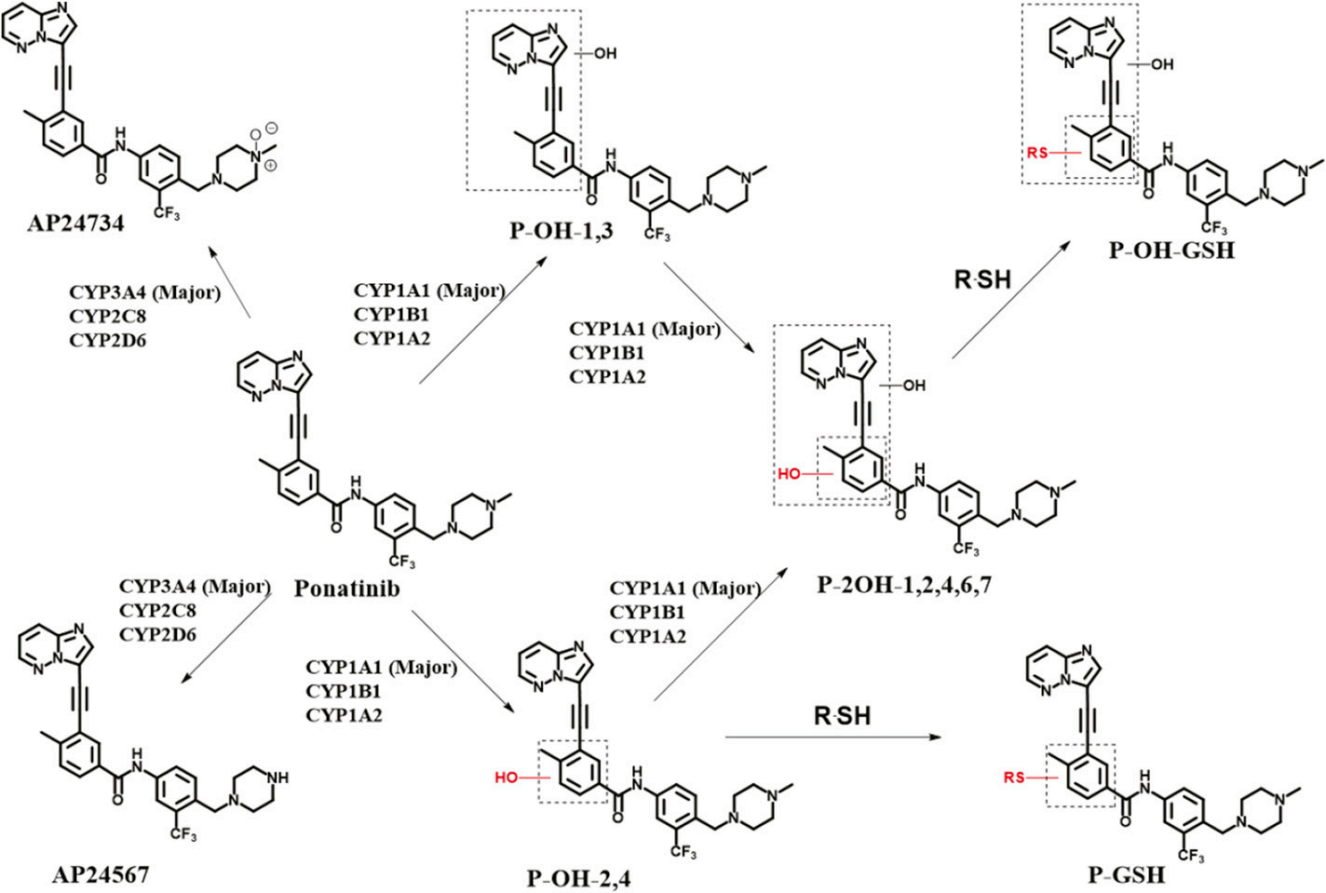
Ponatinib structure and disposition. The structure of ponatinib is shown, along with its known metabolites and pathways of disposition. The involvement of human P450 isoforms is also indicated.

Ponatinib is used to treat CML patients who have relapsed following imatinib therapy (Holyoake and Vetrie, 2017). Although effective, the use of this drug is compromised by a number of serious side effects including vascularitis, pancreatitis, and hepatotoxicity (Cortes et al., 2013; Lipton et al., 2016). To obtain greater insights into pathways of ponatinib metabolism and toxicity we investigated the enzymes involved in its disposition and the metabolites produced by different human P450 enzymes (Fig. 5). Studies were carried out in vitro using humanized mouse models. In agreement with the published literature, CYP3A4 was the major hepatic P450 enzyme involved in the production of the *N*-desmethyl (AP24576) and *N*-oxide (AP24734) metabolites (Narasimhan et al., 2013). However, a number of different metabolites were produced by CYP1A1. Surprisingly, this potentially important pathway of ponatinib metabolism has, to our knowledge, not been previously reported. The metabolites produced by CYP1A1 were reduced by the addition of GSH to the incubation medium, suggesting that reactive electrophilic products had been formed, probably epoxides. Epoxides can be inactivated by a number of enzymes, including GSTs and epoxide hydrolases (Seidegård and Ekström, 1997). In support of epoxide formation, we have shown that the formation of the GSH conjugate of ponatinib is greatly increased by the addition of both murine and human GST. In particular, we have demonstrated that Gstp has significant activity in this regard. If an epoxide was the electrophilic intermediate, it might have been anticipated that two GSH adducts would have been formed. This may be the case, but not observed, if the two adducts were not separated by ultra-performance LC. Also, the MS fragmentation pattern would unfortunately not distinguish the sites of glutathionylation. In addition, it is feasible that one site of glutathionylation was preferred due to steric differences, or the reaction catalyzed by Gstp generates a site-specific product. The formation of reactive epoxides from ponatinib and their inactivation by Gstp has a number of possible implications. Epoxides are chemically reactive and can react covalently with both DNA and proteins to cause mutations and toxicity (Hinson and Roberts, 1992; Boelsterli, 1993; Swenberg et al., 2011). The nucleophilic sites in proteins include cysteine residues, consistent with our finding here that metabolic activation of ponatinib resulted in cysteine conjugates of Gstp1. The cysteine residues, especially Cys-47 of human enzyme GSTP1-1, have been found to be targeted by many electrophilic compounds resulting from P450 metabolism such as acetaminophen, clozapine, and troglitazone (Orton and Liebler, 2007; Tew, 2007; Boerma et al., 2011, 2012; Zhang et al., 2014). When ponatinib was incubated with Gstp1 in the presence of recombinant human CYP1A1, adducts of ponatinib were observed on Cys-14 and Cys-47. The fact that no adducts of ponatinib to Cys-169 were observed in this study may be due to its low reactivity, or perhaps more likely to the relative insensitivity of our methodology in detecting Cys-169-containing tryptic peptides. It would have been interesting to establish whether the addition of epoxide hydrolase to the incubation reduced the formation of the GSH conjugate. However, we have been unable to source any functionally active epoxide hydrolase, and while we believe that epoxide formation is the initial oxidation step, this remains to be formally established.

Therefore, it is possible that the formation of reactive epoxides from ponatinib contributes to the side effects associated with its clinical use. CYP1A1 is a highly inducible enzyme and unlike many other P450s can be expressed in most tissues (Hussain et al., 2014). Metabolic activation could therefore, in principle, occur in all of the reported ponatinib target tissues. CYP1A1 levels are constitutively very low, but are highly inducible on activation of the aryl hydrocarbon receptor by compounds including polycyclic aromatic hydrocarbons found in cigarette smoke (Schiwy et al., 2015). It is interesting to note that hypertension or vaso-occlusive disease observed following ponatinib administration has been associated with smoking (Jain et al., 2015). This potential association is therefore worthy of further epidemiologic studies. If reactive metabolites of ponatinib are involved in its toxicity this could be reduced using inhibitors of CYP1A1. It is worthy of note that the reactive metabolites of ponatinib are also inactivated by conjugation with GSH, mediated at least in part by GSTP. GSTP is expressed in most cell types and indeed in many tumors resistant to anticancer drugs (Henderson et al., 2011, 2014; Tew and Townsend, 2011). Therefore, cellular GSH and GSTP status may also be an important determinant of ponatinib side effects.

In addition to possible involvement in ponatinib side effects, or indeed as a determinant of intratumoral drug concentration, CYP1A1 and CYP1A2 may also contribute to ponatinib exposure since plasma levels and half-life were significantly changed in CYP1A1/1A2 humanized mice treated with aryl hydrocarbon receptor activators (Fig. 4). The PK parameters obtained using this model showed comparable plasma half-life (*t*_1/2_, 13.9 hours) to that in human (∼24 hours), whereas the *t*_1/2_ value was 6.2 hours in WT mice demonstrating the strengths of using humanized models for such studies.

CYP1 family enzymes, especially CYP1A1 and CYP1B1, are overexpressed in myeloblastic and lymphoid cell lines such as U937, BALL-1, and HL-60 (Nagai et al., 2002). The potential expression of these enzymes in CML cells may also be a factor in drug efficacy, or indeed drug resistance. Indeed, our findings provide a mechanistic rationale for the recent report that CML patients that are smokers are at increased risk of disease progression and premature death (Lauseker et al., 2017).

## Abbreviations

AUC: area under the curve
CML: chronic myeloid leukemia
GSH: glutathione
GST: glutathione *S*-transferase
GSTP: glutathione *S*-transferase P
LC: liquid chromatography
3-MC: 3-methylcholanthrene
mGstp1: mouse glutathione *S*-transferase p1
MRM: multiple reactions monitoring
MS: mass spectrometry
P-GSH: ponatinib-glutathione
PK: pharmacokinetic
P450: cytochrome P450
TCDD: 2,3,7,8-tetrachlorodibenzodioxin
WT: wild type
